# Supplementary material for: Sorting at embryonic boundaries requires high heterotypic interfacial tension
Source: Nat Commun. 2017 Jul 31;8:157. doi: 10.1038/s41467-017-00146-x (PMC5537356; doi:10.1038/s41467-017-00146-x)
Supplement: Supplementary file 2 — Supplementary Software 1 [file 41467_2017_146_MOESM2_ESM.zip › PottsModel/SrcPottsModel/doc/model/package-frame.html]

model


# model

## Interfaces

- Coordinates
- EnergyFunction
- IEnergyFunction
- IPositionManager
- KnowsConstants
- Vector

## Classes

- AreaEnergyFunction
- CartesianCoordinates
- Cell
- CellSet
- Constants
- EnergyManager
- FiniteVector
- HalfLine
- HexagonPositionManager
- InterfacialTensionEnergyFunction
- Lattice
- LatticeUtilities
- Pair
- PerimeterEnergyFunction
- Pixel
- PixelInteractionEnergyFunction
- SquareCell
- SquarePositionManager
- VectorTests
- VectorUtils

## Enums

- Cell.CellType
- Cell.Energy
- Cell.State
- Constants.Model
- EnergyFunction.Energy
- IPositionManager.Position
- Lattice.State
- Lattice.Type
- Pixel.PixelType
